# TNFA-863 polymorphism is associated with a reduced risk of Chronic Obstructive Pulmonary Disease: A replication study

**DOI:** 10.1186/1471-2350-12-132

**Published:** 2011-10-10

**Authors:** Elizabeth Córdoba-Lanús, Rebeca Baz-Dávila, Juan P de-Torres, María C Rodríguez-Pérez, Nicole Maca-Meyer, Nerea Varo, Chaxiraxi Medina-Coello, Armando Aguirre-Jaime, Ciro Casanova

**Affiliations:** 1 Research Unit, Hospital Universitario Nuestra Señora de Candelaria, Santa Cruz de Tenerife, Spain; 2 Pulmonary Department, Hospital Universitario Nuestra Señora de Candelaria, Santa Cruz de Tenerife, Spain; 3 Pulmonary Department, Clínica Universidad de Navarra, Spain; 4 Biochemical Analysis Department, Clínica Universidad de Navarra, Spain

## Abstract

**Background**

TNF-α mediated inflammation is thought to play a key role in the respiratory and systemic features of Chronic Obstructive Pulmonary Disease. The aim of the present study was to replicate and extend recent findings in Taiwanese and Caucasian populations of associations between COPD susceptibility and variants of the *TNFA *gene in a Spanish cohort.

**Methods**

The 3 reported SNPs were complemented with nine tag single nucleotide polymorphisms (SNP) of the *TNFA *and *LTA *genes and genotyped in 724 individuals (202 COPD patients, 90 smokers without COPD and 432 healthy controls). Pulmonary function parameters and serum inflammatory markers were also measured in COPD patients.

**Results**

The *TNFA *rs1800630 (-863C/A) SNP was associated with a lower COPD susceptibility (ORadj = 0.50, 95% CI = 0.33-0.77, p = 0.001). The -863A allele was also associated with less severe forms of the disease (GOLD stages I and II) (ORadj = 0.303, 95%CI = 0.14-0.65, p = 0.014) and with lower scores of the BODE index (< 2) (ORadj = 0.40, 95%CI = 0.17-0.94, p = 0.037). Moreover, the -863A carrier genotype was associated with a better FEV_1 _percent predicted (p = 0.004) and a lower BODE index (p = 0.003) over a 2 yrs follow-up period. None of the *TNFA *or *LTA *gene variants correlated with the serum inflammatory markers in COPD patients (p > 0.05).

**Conclusions**

We replicated the previously reported association between the *TNFA *-863 SNP and COPD. *TNFA *-863A allele may confer a protective effect to the susceptibility to the disease in the Spanish population.

## Background

Tumour necrosis factor alpha (TNF-α) is critical in the regulation of inflammation by inducing a cascade of other inflammatory cytokines, chemokines, and other growth factors [1]. The results of several *in vitro *and *in vivo *investigations indicate that high-level generation of TNF-α leads to an exacerbation of the inflammatory and pro-oxidative responses that are important in the pathogenesis of many diseases. TNF-α mediated inflammation is thought to play a key role in both the respiratory and systemic features of Chronic Obstructive Pulmonary Disease (COPD) [2].

Higher concentration of TNF-α has been reported in induced sputum in stable COPD patients and during exacerbations. This cytokine was also found increased in bronchial biopsies and bronchoalveolar lavage fluid of COPD patients when compared with control subjects [3]. A meta-analysis study found higher circulating TNF-α levels in COPD patients when compared to controls [4] and these were also related with important weigh loss in severe patients [5].

Genetic variation in the promoter region of *TNFA *(chromosome 6p21) has been associated with different phenotype expressions, and a wide range of autoimmune, infectious, and oncologic diseases [2]. A common G-to-A single nucleotide polymorphism (SNP) at position -308 (rs1800629), directly affects gene regulation and has been associated with increased transcriptional activity of *TNFA *in various disorders [6,7]. This polymorphism has been studied in several COPD related phenotypes, with contradictory results. It has also been associated with chronic bronchitis and an increased risk of airflow obstruction in Asian populations [8,9]. Most studies in Caucasian populations could not demonstrate this association [10-13]. Only two studies showed an association of the -308 SNP and COPD, the first being a large family study from the Boston early-onset COPD study (2006) and the second performed by Gingo and colleagues (2008) found an increased risk of the A allele of being associated with the disease [14,15].

A 2010 study performed in Taiwanese individuals identified *TNF *-863A (rs1800630) variant to be associated less frequently with COPD [16].

The neighbouring gene, lymphotoxin alpha (*LTA) *has also been related to alter TNF-α expression. TNF-α and LT-α are proinflammatory cytokines coded for by the *TNFA *and *LTA *genes, respectively, mediating a large variety of inflammatory and immunostimulatory responses. The +252 (A to G) SNP in the *LTA *gene has received attention due to its supposed implication in gene regulation [17] and reported associations with several diseases [18,19]. Higher levels of TNF-α have been reported to be associated with SNPs in the *LTA *gene [20]. A case-control study performed on a Russian population reported +252G allele to be associated with a subgroup of severe COPD patients [21]. There is only one study exploring the association of SNPs in both genes and lung function in smokers with mild-to-moderate airway obstruction [12].

The aim of the present study was to replicate recent findings in Taiwanese and Caucasian populations of associations between COPD susceptibility and variants (rs1800629, rs361525 and rs1800630) of the *TNFA *gene in a Spanish cohort. We further aimed to extend on these findings by assessing whether variants/haplotypes of the entire *TNFA *and *LTA *genes may influence COPD susceptibility, severity and lung function decline in a Spanish cohort.

## Methods

### Subjects

Caucasian individuals included in this study were divided into three groups, COPD patients, smokers without COPD and healthy controls. COPD outpatients were recruited from a pulmonary clinic at the Hospital Universitario N. S. de Candelaria (Canary Islands, Spain) from August 2002 to July 2009. Patients with a wide range of disease severity were included using the following criteria: age > 40 years, smoking history > 10 pack-years and a post-bronchodilator FEV_1_/FVC ratio < 0.70 measured 20 min after the administration of 400 mg of inhaled albuterol. Pulmonary function tests were measured according to GOLD guidelines [22]. The arterial oxygen tension (PaO_2_) was measured at rest. Severe hypoxemia was defined by PaO_2 _values < 60 mmHg. Exercise capacity was also tested using the best of two 6-min walking distance (6MWD) tests separated by at least 30 min following the ATS recommendations [23]. The body mass index (BMI) was calculated as the weight in kilograms divided by the square of the height in meters. The BODE (Body Mass Index, Airflow Obstruction, Dyspnea, Exercise Performance) Index was calculated as previously described [24]. All patients were clinically stable (no exacerbation for at least 2 months) at the time of evaluation. Patients were excluded if they had history of other diseases like asthma or bronchiectasis. As a control group we included 432 individuals without respiratory disease and no smoking history from an ongoing general adult population cohort named CDC project in the Canary Islands [25]. Finally, the third group included 90 current smokers with a cumulative smoking history of > 15 pack-years and normal lung function (FEV_1_% pred > 0.80; FEV_1_/FVC > 0.70). In order to minimize any possible effect of population structure on our estimations, cases and controls were recruited if they have at least two generations of Canarian ancestry [26].

The Ethics Committee of Clinical Investigation of the Hospital approved this study (approval N°36) and written informed consents were obtained from all subjects.

### SNPs selection and genotyping

We selected the haplotype-tagging SNPs in the *TNFA *and *LTA *genes using the resequencing data of the European individuals from the SeattleSNPs database http://pga.gs.washington.edu. A multimarker tagging algorithm with criteria of r2 > 0.8 and minor allele frequency (MAF) ≥ 0.1 were used with the Haploview program (version 3.2) [27]. Eleven SNPs in the *TNFA *gene (GenBank, AY066019) and *LTA *gene (GenBank AY070490) were chosen. *TNFA *-857, -376 and -238 SNPs (rs1799724, rs1800750 and rs361525) were included in the study because they had been reported as susceptibility locus in other diseases [28-30].

Venous blood was collected from each individual and total genomic DNA was extracted using the commercially available GFX Kit (GE Healthcare). All tSNPs selected were genotyped using SNaPshot^® ^SNP Genotyping Assay (Applied Biosystems, Foster City, CA). Genotyping was blind to case or control status of samples. A random 20% of the samples were genotyped in duplicate for quality control. The concordance rate for the duplicate samples was 100%. Moreover, as a confirmational method a random number of samples (patients and control subjects) was genotyped by direct sequencing on an ABI PRISM 310 Genetic Analyser (Applied Biosystems). Samples not yielding the genotypes of all SNPs were excluded from analysis; so 721 individuals remained in the study (199 COPD cases).

### Serum measurements

COPD patients' serum was separated from whole blood by centrifugation (3200 rpm) and aliquots were stored at -80°C until laboratory analysis. Circulating levels of IL-6, IL-8, IL-16, TNF-α, MCP-1, MMP-9, PARC and VEGF were measured by ELISA (R&D System, Minneapolis) according to manufacter's instructions. The detection limits were 0.7 pg/ml, 3.5, 6.2, 5.0, 156, 10, and 5.0, respectively. The within-assay coefficient of variation for all assays was less than 10%.

### Statistical analysis

Demographic and main clinical data are presented as percentage and mean ± SD. Differences among groups were explored using Student *t *test, Mann-Whitney *U *rank test, or Pearson χ2 test as appropriate. To perform the analysis we used the statistical package SPSS v17 (SPSS, Inc). Evaluation of the progression of the disease of clinical and pulmonary function variables using the SNPs as comparative factor was performed using a general linear modelling for repeated measures (GLIM). For analysis of severity, GOLD stage and BODE index were dichotomised to a mild-to-moderate (GOLD stage I-II and BODE < 2) and severe disease (GOLD stage III-IV and BODE ≥ 3) (table 1). Allele and genotype distribution in the patients and control groups was compared using contingency tables and χ^2 ^test for independence. Disequilibrium values, departures from Hardy-Weinberg equilibrium and haplotype reconstruction from biallelic polymorphisms, were calculated using the GENEPOP v3.4, Haploview v3.2 and PHASE v2.1 software programmes [29-32] as appropriate. A generalized additive model was first used to estimate the genotype relative risks (OR and their 95% confidence intervals) for COPD susceptibility. This was calculated by using SNPSTATS [33]. The effective number of independent marker loci, based on pairwise LD between genotyped SNPs for populations independently, was used for adjustment for multiple tests [34]. To test haplotype associations and for checking for consistency of the inferred data, haplotypes were reconstructed from the best average goodness-of-fit output of six PHASE 2.1 runs with 1,000 permutations [32]. Multivariate logistic regression analysis was used to adjust for potential risk and confounding factors (age, sex, pack-yrs of smoking). ANOVA was performed to test the effect of the polymorphisms and serum proteins levels. In every case, a two-sided p value of < 0.05 was considered statistically significant.

**Table 1:** Demographic characteristics of COPD patients, smokers and healthy individuals included in the association study

Variable	Cases (n = 202)	Smoking controls (n = 90)	Healthy controls (n = 432)	P-value
**Gender (male %)**	76	49	75	< 0.0001
Age (years)*	62 ± 10	48 ± 9	53 ± 8	< 0.0001
Smoking history(pack-yrs)^†^*	63 ± 27	35 ± 18	__	< 0.0001
**FEV**_1_*	1.53 ± 0.7	3.06 ± 0.71	__	< 0.0001
**FEV**_**1 **_% pred*	56 ± 22	102 ± 15	__	< 0.0001
FVC % pred*	86 ± 23	108 ± 16	__	< 0.0001
**FEV**_**1**_/FVC*	52 ± 12	79 ± 6	__	< 0.0001
6MWD (m) *	490 ± 86	__	__	
BMI (Kg/m^2^)*	27 ± 5	__	__	
BODE index*	2 ± 0.7	__	__	
**BODE index < 2**^**‡**^	111	__	__	
**BODE index ≥ 3**^**‡**^	52	__	__	
**GOLD I-II**^**‡**^	119	__	__	
**GOLD III-IV**^**‡**^	83	__	__	

*Data are presented as mean ± SD

^† ^Number of packs of cigarettes smoked per day × number of years smoking

^‡ ^Number of subjects in the two groups of GOLD and BODE index considered to analysis.

FEV_1_: forced expiratory volume in one second; FVC: forced expiratory volume; % pred: per cent predicted; 6MWD: six-min walk distance test; BMI: body mass index.

## Results

Table 1 summarizes the demographic data and baseline characteristics of the study groups. Of the 202 stable COPD patients (153 men and 49 women), 59% of patients were in GOLD stages I-II and 41% in GOLD stages III-IV. Similarly, of the 163 patients where the BODE index was possible to calculate, 68% scored < 2 in the BODE index and 32% scored higher (BODE ≥ 3).

**Figure 1:**
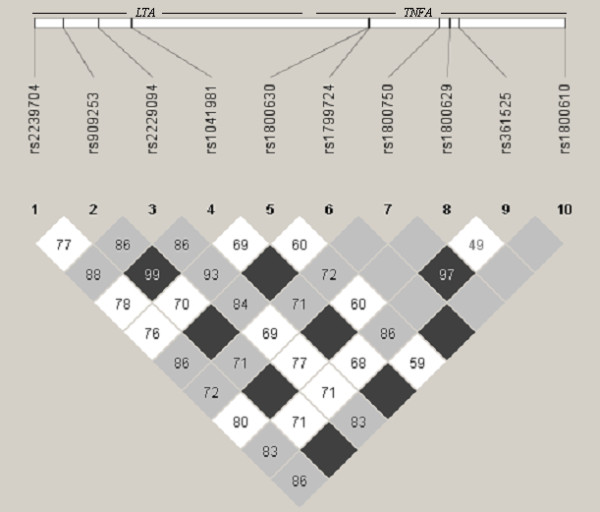
**Pairwise linkage disequilibrium between SNPs in the *LTA *and *TNFA *genes**. Values provided in each box, were estimated as r^2 ^using Haploview 3.2. Dark grey regions depict strong LD (1.0), pale grey and white regions represent low LD.

**Figure 2:**
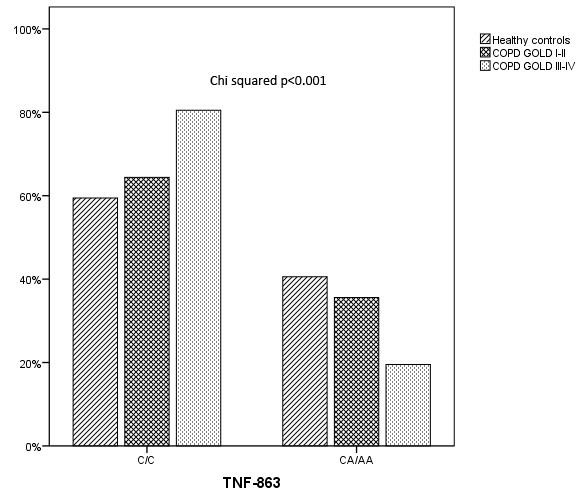
**Frequency distribution of the *TNFA *-863 CC and CA+AA genotypes (dominant model) in three groups: healthy control subjects, GOLD I-II and GOLD III-IV COPD patients**.

All SNPs conformed to HWE with the exception of rs746868 (p < 0.05), which was then excluded from further analyses. The values for the linkage disequilibrium (LD) between the SNPs in the *TNFA* and *LTA *genes are shown in Figure 1. Very strong levels of LD can be observed between SNPs within both genes. SNP -863C/A in the *TNFA *gene was significantly associated with COPD susceptibility as previously reported [16]. The frequency of the -863 A carrier genotype was significantly lower in COPD patients when compared with healthy controls yielding an odds ratio of 0.61 (95% CI = 0.42-0.88, p = 0.008) under a dominant model of inheritance (see Additional file 1). This result remained significant after adjusting for possible confounding factors as age and gender (OR = 0.50, 95% CI = 0.33-0.77, p = 0.001) in a multivariate analysis (table 2). The -863A allele resulted associated with less severe forms of the disease (GOLD stages I and II) when compared with severe COPD (GOLD stages III and IV) (ORadj = 0.303, 95%CI = 0.14-0.65, p = 0.014) (Figure 2). This was consistent with the finding that the -863A allele was significantly associated with patients who scored lower in the BODE index (BODE < 2 vs. BODE ≥ 3; ORadj = 0.40, 95%CI = 0.17-0.94, p = 0.037) (table 3). Moreover, when analysing longitudinal clinical data of the patients cohort, it resulted that the -863 SNP was significantly associated with FEV_1 _percent predicted (FEV_1 _% pred) (p = 0.004) and the BODE index (p = 0.003) over the 2 yrs follow-up period. The relation found between these clinical parameters and the -863 genotype did not vary among these groups over time (p > 0.05). In addition we found that FEV_1 _% pred and the BODE index differed significantly in each one of three moments included in the longitudinal analysis (table 4).

**Table 2:** Association of SNPs in the *LTA *and *TNFA *genes with Chronic Obstructive Pulmonary Disease (COPD)

			COPD cases vs. healthy control			
Gene position	SNP ID	Seattle SNP database	COPD cases MAF %	Healthy controls MAF %	**OR_adj _(95% CI) **^a^	P-value*
***LTA***						
+49(C/A)	rs2239704	Lt_2202	0.42	0.39	1.05 (0.79-1.4)	0.73
+252(A/G)	rs909253	Lt_2374	0.30	0.30	1.08 (0.79-1.46)	0.63
+495(T/C)	rs2229094	Lt_2619	0.31	0.33	0.83 (0.62-1.11)	0.2
+720(C/A)	rs1041981	Lt_2847	0.29	0.30	1.05 (0.77-1.42)	0.78
***TNFA***						
-863(C/A)	rs1800630	Lt_4539	0.16	0.22	0.58 (0.41-0.83)	**0.002**
					0.50 (0.33-0.77)	0.001**
-857(C/T)	rs1799724	Lt_4545	0.09	0.09	1.41 (0.88-2.27)	0.16
-376(G/A)	rs1800750	TNF_214	0.04	0.03	1.30 (0.64-2.66)	0.47
-308(G/A)	rs1800629	TNF_282	0.15	0.15	0.97 (0.66-1.42)	0.87
-238(G/A)	rs361525	TNF_352	0.06	0.06	1.25 (0.72-2.19)	0.43
+489(G/A)	rs180610	TNF_1078	0.09	0.09	1.42 (0.88-2.22)	0.15

Data are presented as MAF: minor allele frequency (%). OR_adj_: odds ratio; CI: confidence interval. *p values < 0.05. Age and gender were included in a multivariate logistic regression analyses as potential confounding factors in an additive model. **p value from a dominant genetic model.

**Table 3:** Association tests of *TNFA *-863 C/A polymorphism with COPD using multiple logistic regression analysis

	BODE < 2 vs BODE ≥ 3			
**Included variable**^**a**^	**B**^**b**^	**OR**_**adjusted**_	**95% CI**	**P-value**

*Dominant model*				
CA+ AA	-9.17	0.400	0.17-0.94	0.037*
Age	-0.023	0.977	0.94-1.02	0.256
Gender	-0.162	0.850	0.38-1.94	0.699
Smoking (pack/yr)	1.571	4.811	0.99-1.02	0.305

^a^Age, gender and smoking history (pack/years) were included in the analyses as confounding variables, ^b^Regression coefficient, OR: odds ratio; CI: confidence interval, *p values < 0.05

**Table 4:** Longitudinal association of *TNFA *-863 polymorphism with pulmonary function and BODE index in COPD patients

Parameter	SNP Genotype	Time			**P-value**^†^
			
		baseline	1° year	2° year	
FEV_1 _% pred	CC	55.2 ± 20.1	52.1 ± 18.7	49.1 ± 19.7	**0.004**
	CA+AA	64.9 ± 21.6	61.3 ± 19.4	62.0 ± 17.1	
	**P-value**	**0.003**	**0.017**	**0.002**	

BODE	CC	2 (0-5)	2(0-6)	2(0-6)	**0.003**
	CA+AA	1 (0-4)	1(0-4)	1(0-4)	
	**P-value**	**0.001**	**0.006**	**0.016**	

Data are presented as mean **± **standard deviation (X ± SD) for FEV_1 _% pred and compared by T-Student test. The Bode index was summarized as median (5^th^-95^th ^pc) and compared by U-Mann Whitney test. ^† ^Global p-value for GLIM test for repeated measures inter-groups.

The obtained p-values were corrected for multiple testing by the effective number of independent tests (8.85) resulting in a significant association for allele A carriers of the -863 SNP between the COPD cases and the healthy controls group (adjusted p = 0.02). No significant differences were found in COPD patients versus smokers without the disease when analysing single polymorphisms.

Global tests for haplotype were predicted from genotypic data. These analyses revealed no differences in frequency distribution between cases and the controls groups: cases vs. smokers without the disease (p = 0.54) and cases vs. healthy controls (p = 0.35) (table 5). When we calculated haplotype relative effect to the most common haplotype no increased risk/susceptibility was observed for the COPD patients sample. However, the haplotype that contains the -863A allele was less frequently observed in COPD cases than in healthy controls individuals (non significant). In contrast, no association was found with the previously reported [14,15] SNPs rs1800629, rs361525 in *TNF *gene.

**Table 5:** Haplotype frequency of the *LTA *and *TNFA *genes SNPs in COPD patients vs. Healthy control individuals

	Haplotypes*	**Total freq**.	Healthy controls n = 432	COPD cases n = 199	ORadj (95%CI)ª	p-value
1	A A T C C C G G G G	0.2827	0.2806	0.2982	1.00	
2	C A C C A C G G G G	0.1703	0.1815	0.1461	0.70 (0.45 - 1.08)	0.11
3	C G T A C C G A G G	0.1311	0.1328	0.1220	1.03 (0.64 - 1.66)	0.90
4	C G T A C C G G G G	0.1268	0.1280	0.1257	1.09 (0.67 - 1.78)	0.72
5	C A C C C C G G G G	0.0806	0.0723	0.0944	1.27 (0.72 - 2.23)	0.40
6	A A T C C T G G G A	0.0762	0.0749	0.0781	1.33 (0.76 - 2.35)	0.32
7	C A C C C C A G A G	0.0251	0.0205	0.0327	1.74 (0.75 - 4.00)	0.19
8	C A C C C C G G A G	0.0202	0.0196	0.0212	1.54 (0.59 - 4.02)	0.38
9	C A T C C C G G G G	0.0165	0.0177	0.0139	1.59 (0.56 - 4.48)	0.38
10	Rare	0.0703				

*Haplotypes: LT+49, LT+252, LT+495, LT+720, TNF-863, TNF-857, TNF-376, TNF-308, TNF-238, TNF+489.

ª ORadj: odds ratio adjusted by age and gender; CI: confidence interval.

None of the *TNFA *or *LTA *gene variants analysed in the present study resulted correlated with the circulating IL-6, IL-8, IL-16, TNF-α, MCP-1, MMP-9, PARC or VEGF levels in serum of COPD patients (p > 0.05, data not shown).

## Discussion

Our objective was to replicate results from previous studies [14-16] showing association between *TNF *polymorphisms and COPD. The present study investigates the association of *TNFA *and *LTA *genes polymorphisms and COPD presence and severity including eleven SNPs of two genes that cover most of their variability. The most important finding of our research suggests that the previously reported [16]*TNFA *-863A allele is less frequent in COPD patients. The presence of this allele was associated with a less severe form of the disease (GOLD stages I-II) and with lower scores of the BODE index (< 2). Moreover, this association remained unchanged after adjustment for confounding factors like age and gender, suggesting a robust effect.

The most recent study, performed in Taiwanese individuals, found that the -863A allele was associated less frequently with COPD, with an increased FEV1/FVC ratio and higher BMI among heavy smokers [16]. Another two studies on COPD found an association of the haplotype containing the -863A variant with a lower risk for susceptibility to COPD, one performed on a Greek population [35] and the second performed on a Caucasian one [14]. Similarly, in our study the only haplotype that contains the -863A allele also tended to be less likely in COPD patients. This region of the *TNFA *promoter seems to affect transcription factors binding capacity. The variant -863 has been reported to specifically reduce 10 fold the binding affinity of the transcription nuclear factor (NF-κB) specially the form p-50-p50 [36]. Most of the inflammatory proteins that are up regulated in airways of COPD patients are regulated by the transcription nuclear factor NF-κB that is activated in alveolar macrophages of COPD patients [37]. The -863A allele has been reported associated with elevated TNF-α production by peripheral blood mononuclear cells stimulated with concavalin A [38] while others have associated this variant with reduced circulating levels of the cytokine [30,39]. In the present analysis we could not find any relation between the SNP and serum levels of TNF-α or any other inflammatory cytokine measured.

One strength of the present study is that the association found for the -863 SNP and FEV_1 _and BODE index was also replicated when we explored the SNP association in the three longitudinal clinical measures registered over the two years follow up period. This is the first report of a case-control study that evaluated the influence of gene variants on clinical and pulmonary function longitudinal data in a cohort of COPD patients.

However, we did not replicate other previously reported [14,15] associations of *TNF *polymorphisms with the disease. Consistent with the majority of previous studies on Caucasians we found that the most studied *TNFA *polymorphism (-308G/A) was not associated with the presence of COPD in our sample. Only two studies on Caucasians found significant association between this SNP and COPD presence. The first was a family based research from the Boston Early Onset COPD study [15] that analysed 17 families but these findings could not be replicated in a case control study of later onset COPD patients. The second, a case-control study [14], found that the -308A allele had a higher risk of being associated with COPD and was also associated with worse FEV1/FVC. In that study the authors included as cases a subgroup of 11 individuals with emphysema that did not met COPD diagnosis criteria, and therefore this subgroup may have influence the final results. In addition, a meta-analysis study performed by Smolonska and colleagues [40] reported that *TNFA *was found associated with COPD susceptibility only in Asians.

In relation to other SNPs within the two genes analysed in previous studies, none of them were found associated with COPD in the present study. This finding is supported by the results obtained by the majority of researches performed in Caucasian individuals [10-15,40].

The present study has several limitations. Firstly, the reduced sample size of the smoking control group without the disease cannot exclude that the lack of association found between cases and smokers without COPD with the -863 SNP could be a false negative result due to a type II error. The present analysis should be tested in a large sample of smoking controls. Second, population stratification should be addressed in this kind of studies. Even though, it is known that human populations from Spain, including Canary Islands, are highly homogenous in their genetic background [26], in order to reduce the possibility of subtle population stratification we included 100% Canarian individuals in our control groups (smokers and non-smokers) with at least two generations of ancestors from the islands. And third, we have not find correlation between the genetic variants analysed and the functional aspects of the progression of the disease probably due to the short observational period of our study. Mayor long-term longitudinal studies are needed in order to confirm the relationships of certain factors with clinical and functional variables of the disease.

We have found that COPD patients with the variant -863A presented better lung function (GOLD stages I-II). The -863A allele may be conferring a protective effect on the progression of the disease, which could be explained by the fact that COPD patients progress in different manner along time. It has been hypothesized that the disease progression could be represented by three steps suggesting that, in most smokers the disease process will not advance if innate inflammation is minimized which situate individuals at step 1 or 2 of the model, which is comparable to smokers with normal lung function or COPD patients at GOLD stage I and II respectively [41]. This may be the consequence of a genetic background that protects smokers in progressing to severe forms of the disease as it is shown by our results.

## Conclusion

Our data confirm a previous study where the minor allele of SNP -863 in the *TNFA *gene was associated with a reduced risk of COPD. Our findings also suggest that the-863A allele is associated with less severe forms of the disease. No association was observed for other SNPs in *TNF-LT *genes and COPD. This is the first study of *TNFA *and *LTA *gene polymorphisms in COPD performed in a Spanish Population.

## Abbreviations

ATS: American Thoracic Society; BMI: Body Mass Index; BODE: Body Mass Index; Airflow Obstruction; Dyspnea; Exercise Performance; CI: Confidence Intervals; COPD: Chronic Obstructive Pulmonary Disease; FEV_1: _Forced Expiratory Volume in one second; FVC: Forced Vital Capacity; MAF: Minor Allele Frequency; LTA: Lymphotoxin Alpha; OR: Odds Ratio; PaO_2: _Arterial Oxygen Tension; SNP: Single Nucleotide Polymorphism; 6MWD: 6-Min Walking Distance; TNFA: Tumour Necrosis Factor Alpha; tSNPs: Tag Single Nucleotide Polymorphism.

## Competing interests

The authors declare that they have no competing interests.

## Authors' contributions

Conceived and designed the study: ECL, CC. Performed the experiments: ECL, RBD, CMC, NV. Enrolled patients: CC, JPT. Enrolled control subjects: CRP. Analysed the data: ECL, RBD, and AAJ. Wrote the paper: ECL, CC, RBD, and JPT. All authors read and approved the final manuscript.

## Pre-publication history

The pre-publication history for this paper can be accessed here:

http://www.biomedcentral.com/1471-2350/12/132/prepub

## Supplementary Material

Additional file 1 **Table 6. Genotype frequency distribution of the *LTA *and *TNFA *genes SNPs in COPD patients, healthy control and smoking control individuals**. Click here for file
