# Supplementary material for: TNFA-863 polymorphism is associated with a reduced risk of Chronic Obstructive Pulmonary Disease: A replication study
Source: BMC Med Genet. 2011 Oct 10;12:132. doi: 10.1186/1471-2350-12-132 (PMC3209447; doi:10.1186/1471-2350-12-132)
Supplement: Additional file 1 — Table 6. Genotype frequency distribution of the LTA and TNFA genes SNPs in COPD patients, healthy control and smoking control individuals. [file 1471-2350-12-132-S1.DOC]

Table 6. Genotype frequency distribution of the *LTA* and *TNFA* genes SNPs in COPD patients, healthy control and smoking control individuals.

| Genotype | **All subjects**  N=631 | | **Healthy controls**  n=432 | | **Smoking controls**  N=90 | | **COPD cases**  n=199 | |
| --- | --- | --- | --- | --- | --- | --- | --- | --- |
|  | No. | Freq. | No. | Freq. | No. | Freq. | No. | Freq. |
| Lt +49 rs2239704 |  |  |  |  |  |  |  |  |
| C/C | 222 | 0.35 | 158 | 0.37 | 33 | 0.37 | 64 | 0.32 |
| C/A | 310 | 0.49 | 209 | 0.48 | 43 | 0.48 | 101 | 0.51 |
| A/A | 99 | 0.16 | 65 | 0.15 | 14 | 0.16 | 34 | 0.17 |
| LT +252 rs909253 |  |  |  |  |  |  |  |  |
| A/A | 312 | 0.49 | 218 | 0.5 | 44 | 0.49 | 94 | 0.47 |
| A/G | 265 | 0.42 | 173 | 0.4 | 40 | 0.44 | 92 | 0.46 |
| G/G | 54 | 0.09 | 41 | 0.09 | 6 | 0.07 | 13 | 0.07 |
| Lt +495 rs2229094 |  |  |  |  |  |  |  |  |
| T/T | 288 | 0.46 | 193 | 0.45 | 41 | 0.46 | 95 | 0.48 |
| T/C | 280 | 0.44 | 194 | 0.45 | 43 | 0.48 | 86 | 0.43 |
| C/C | 63 | 0.1 | 45 | 0.1 | 6 | 0.07 | 18 | 0.09 |
| Lt +720 rs1041981 |  |  |  |  |  |  |  |  |
| C/C | 311 | 0.49 | 216 | 0.5 | 44 | 0.49 | 95 | 0.48 |
| C/A | 267 | 0.42 | 175 | 0.41 | 39 | 0.43 | 92 | 0.46 |
| A/A | 53 | 0.08 | 41 | 0.09 | 7 | 0.08 | 12 | 0.06 |
| TNF -863 rs1800630 |  |  |  |  |  |  |  |  |
| C/C | 410 | 0.65 | 266 | 0.62 | 61 | 0.68 | 144 | 0.72 |
| C/A | 188 | 0.3 | 141 | 0.33 | 28 | 0.31 | 47 | 0.24 |
| A/A | 33 | 0.05 | 25 | 0.06 | 1 | 0.01 | 8 | 0.04 |
| TNF -857 rs1799724 |  |  |  |  |  |  |  |  |
| C/C | 528 | 0.84 | 362 | 0.84 | 75 | 0.83 | 166 | 0.83 |
| C/T | 95 | 0.15 | 65 | 0.15 | 14 | 0.16 | 30 | 0.15 |
| T/T | 8 | 0.01 | 5 | 0.01 | 1 | 0.01 | 3 | 0.02 |
| TNF -376 rs1800750 |  |  |  |  |  |  |  |  |
| G/G | 591 | 0.94 | 406 | 0.94 | 81 | 0.9 | 185 | 0.93 |
| G/A | 39 | 0.06 | 25 | 0.06 | 9 | 0.1 | 14 | 0.07 |
| A/A | - |  | - |  | - |  | - |  |
| TNF -308 rs1800629 |  |  |  |  |  |  |  |  |
| G/G | 457 | 0.72 | 314 | 0.73 | 68 | 0.76 | 143 | 0.72 |
| G/A | 158 | 0.25 | 105 | 0.24 | 21 | 0.23 | 53 | 0.27 |
| A/A | 16 | 0.03 | 13 | 0.03 | 1 | 0.01 | 3 | 0.02 |
| TNF -238 rs361525 |  |  |  |  |  |  |  |  |
| G/G | 560 | 0.89 | 385 | 0.89 | 75 | 0.83 | 175 | 0.88 |
| G/A | 68 | 0.11 | 44 | 0.1 | 13 | 0.14 | 24 | 0.12 |
| A/A | 3 | 0 | 3 | 0.01 | 2 | 0.02 | 0 | 0 |
| TNF +489 rs180610 |  |  |  |  |  |  |  |  |
| G/G | 529 | 0.84 | 363 | 0.84 | 74 | 0.82 | 166 | 0.83 |
| G/A | 94 | 0.15 | 64 | 0.15 | 15 | 0.17 | 30 | 0.15 |
| A/A | 8 | 0.01 | 5 | 0.01 | 1 | 0.01 | 3 | 0.02 |
